# Effects of art therapy on psychological outcomes among children and adolescents with cancer: a systematic review and meta-analysis

**DOI:** 10.1186/s12906-025-04866-2

**Published:** 2025-04-23

**Authors:** Shishuang Zhou, Huiyuan Li, Yuan Yang, YiShu Qi, Weiwei Liu, Lin Mo, Cho Lee Wong

**Affiliations:** 1 The Nethersole School of Nursing, Faculty of Medicine, The Chinese University of Hong Kong, Hong Kong, China; 2 School of Nursing, Army Medical University, Chongqing, China; 3 Department of Outpatient Children’s Hospital, Research Center for Child Health and Disorders, Ministry of Education Key Laboratory of Child Development and Disorders, Chongqing Medical University, National Clinical, Chongqing, China; 4 International Science and Technology Cooperation Base of Child Development and Critical Disorders, Chongqing, China

**Keywords:** **C**hildren and adolescents with cancer, Pediatric cancer, Art therapy, Psychological outcomes, Systematic review

## Abstract

**Background:**

The increasingly rising incidence of cancer among children and adolescents has led to notable psychological challenges for this population. Art therapy, classified within the realm of complementary and alternative medicine interventions and psychotherapy, demonstrates promising potential psychological benefits for children and adolescents. Therefore, a systematic review was conducted to determine the effects of art therapy on improving psychological outcomes among patients with pediatric cancer and identify the details of art therapy.

**Method:**

A systematic review and meta-analysis was conducted. Ten English language databases, two Chinese databases, and grey literature were searched. Two researchers independently conducted study selection, quality assessment and data extraction. The Generic inverse variance method with random-effects models was applied to do meta-analysis.

**Results:**

Three randomized controlled trials (RCTs) and five quasi-experimental studies with acceptable quality involving 452 participants from five countries were included. Our meta-analysis revealed statistically significant improvement in anxiety, depression among pediatric cancer patients. Narrative findings suggested art therapy could improve the overall psychological symptoms, stress, and anger.

**Conclusion:**

Art therapy can positively improve psychological outcomes, particularly anxiety and depression. However, the evidence is weakened by limited studies and methodological heterogeneity. Additional high-quality RCTs with large samples are warranted to confirm and supplement the existing evidence.

**Trial registration:**

This review was registered in PROSPERO with ID CRD42023477700 on 11 November 2023.

**Supplementary Information:**

The online version contains supplementary material available at 10.1186/s12906-025-04866-2.

## Background

Cancer is the leading cause of death among children and adolescents globally [24]. According to Childhood Cancer International and the World Health Organization (WHO) Global Childhood Cancer, approximately 400,000 children and adolescents (age ≤ 19 years old) are diagnosed with cancer annually [49]. The International Agency for Research on Cancer predicts that an estimated 7.6–13.7 million children and adolescents will be diagnosed with cancer between 2020 and 2050 [7].

Cancer and its treatments can lead to negative impacts on the psychological outcomes of patients with pediatric cancer. Cancer treatments such as chemotherapy, radiation therapy, surgery, and bone marrow transplants result in some cancer-related symptoms and adverse effects, thus impacting their emotions [11, 27, 55]. For example, children and adolescents with cancer feel fear because of the pain caused by some invasive procedures. The life-threatening nature of cancer also creates a sense of gravity, mortality, stigma, and uncertainty of treatments among patients with pediatric cancer [34]. The treatments of cancer typically span an extended duration and necessitate multiple hospitalizations, which leads to a constant awareness of illness in children and adolescents with cancer and restricts their ability to engage in regular social activities [13]. Thus, patients with pediatric cancer commonly experience psychological symptoms such as anxiety, depression, stress, and distress [11, 30, 43].

Non-pharmacological interventions have been widely utilized to manage psychological outcomes among children and adolescents with cancer [8, 10, 38]. Some systematic reviews showed that these interventions include psychotherapy, and complementary and alternative medicine (CAM) interventions [8, 28, 32, 47].

Art therapy, classified within the realm of CAM interventions and psychotherapy, is defined as the therapeutic use of visual art forms such as painting, handcraft like clay work, collage, and sculpture, and it has been gradually applied in patients with pediatric cancer ( ([17, 18, 36]). Art therapy is a specific kind of sensory art therapy that encompasses various modalities like art therapy, music therapy, play therapy, and dance therapy [14, 15]. When a therapeutic approach combines two or more forms of sensory art therapies, it refers to creative arts therapy [33, 37].

Compared with other kinds of sensory art therapies, art therapy offers various visual artistic media, and it has been recognized as an effective communication tool for pediatric individuals [41]. Art therapy can stimulate the brain’s right and left hemispheres, allowing art-makers to tap into their emotions, imagination, and non-verbal expression [5, 23, 45]. Through various visual art forms, children and adolescents with cancer can access and process psychological symptoms affected by cancer and treatments that may be difficult to articulate verbally [5, 9]. Several experimental studies have indicated that art therapy can improve psychological symptoms like anxiety, depression, and stress [2, 17, 18].

Three reviews have been conducted on art therapy for children and adolescents with cancer [5, 36, 53].

However, certain limitations need to be considered. First, these reviews included various types of sensory art therapies rather than focusing solely on art therapy. Second, they relied on narrative synthesis without calculating specific effect sizes or conducting a meta-analysis to determine the effectiveness of art therapy.

Although these three reviews have indicated the potential benefits of different forms of sensory art therapies and creative arts therapy for promoting psychological outcomes in patients with pediatric cancer, the effectiveness of art therapy alone in this context remains to be determined. Therefore, this review will primarily focus on art therapy as a specific form of sensory art therapy to determine its effectiveness by employing meticulous study selection.

## Methods

The Cochrane Handbook for Systematic Reviews of Interventions [25], preferred Reporting Items for Systematic Reviews and Meta-Analysis (PRISMA) guidelines ( http://www.prisma-statement.org/ ), and APA's Meta-Analysis Reporting Methods (MARS) [6] were applied to guide this systematic review. This review was registered in PROSPERO with ID CRD42023477700 on 11 November 2023.

### Information sources and search strategy

The search strategy was guided by the PICO framework (Population, Intervention, Comparison, Outcome). Our research question was: "In children and adolescents with cancer (Population), what is the effect of art therapy (Intervention) compared to usual care or other therapies (Comparison) on psychological outcomes (Outcome)?" We searched for articles in electronic databases, including ten English-language databases, namely, Ovid MEDLINE(R), Embase, Scopus, Web-of-science, AMED (Allied and Complementary Medicine), APA PsycArticles Full Text, APA PsycINFO, CINAHL Ultimate, Cochrane, and Ovid Nursing Database, and two Chinese databases (i.e., CNKI and Wanfang Data). To enhance the sensitivity of retrieving relevant articles, we ensured that the keywords contained 1) the target population (neoplasm or neoplas* or neoplasias or cancer* or oncology* or malignant* or carcinoma or tumor* or tumour*); 2) the age group (children; childhood; pediatric; pediatry; pediatric; adolescent; teen; juvenile); and 3) the intervention method (art therapy* or art making* or paint* or drawing or mandala or art appreciation* or art product* or sculpture or clay). The search strategies and Mesh terms were adjusted accordingly to databases (refer to Supplementary 1 for details). Our search was conducted on 12–17 Oct 2023 and updated on 6–9 Feb 2025. The publication date was not restricted.

### Eligibility criteria

#### Inclusion criteria

The inclusion criteria encompassed the following parameters:

- Populations: Children and adolescents diagnosed with any form of cancer, aged ≤ 19 years, and are under treatment. There were no mental health eligibility criteria for participants.
- Intervention: The intervention was art therapy (e.g., different kinds of visual arts such as painting and handcraft like clay, collage, and sculpture; as long as visual art elements are incorporated into the therapeutic process, the specific methods or techniques used can vary).
- Control: The comparison group could be usual care, waitlist, or other therapies.
- Outcomes: Psychological outcomes include psychological symptoms such as anxiety, depression, stress, and anger, as well as positive emotions such as happiness, hope, love, self-efficacy, self-esteem, and psychological well-being.
- Studies: RCTs and quasi-experimental studies with the control group.
- Published languages: English and Chinese.

#### Exclusion criteria

Findings reported as abstracts, conference papers, books, chapters, reviews, clinical correspondence and case reports, and study protocols, or the intervention comprised other sensory art therapies such as music therapy, dance movement, or creative arts therapy, will be excluded.

### Study selection

All researched articles were imported into the literature management software NoteExpress. After removing duplicates, two researchers (ZS and YY) independently conducted a meticulous selection of studies, adhering to predetermined inclusion and exclusion criteria. The evaluation process consisted of an initial assessment of the title and thorough examination of the abstract and full text. A third researcher (QY) resolved any discrepancies and made the final decision.

### Methodological quality assessment

The quality of the included studies was assessed by Cochrane’s risk-of-bias assessment tools: revised Cochrane risk-of-bias tool for randomized trials (ROB 2) and the Risk of Bias in Non-randomized Studies-of Interventions (ROBINS-I). ROB 2 [42] was used to assess the randomized study, which included five domains: bias arising from the randomization process, bias due to deviations from the intended intervention, bias due to missing outcome data, bias in the measurement of the outcome, and bias in the selection of the reported result. ROBINS-I [26] contained seven domains to evaluate the bias due to confounding, selection of participants, deviations from intended interventions, missing data and bias in classification of interventions, measurement of outcomes, and selection of reported results,it was applied to evaluate the quality of quasi-experimental studies. The quality of each included study was determined independently by both researchers (ZS and YY, and a third reviewer (QY resolved any discrepancies. Finally, all three reviewers reached a consensus on the ultimate outcomes of the assessment.

### Data extraction

First, our research team discussed and constructed a standardized extraction form comprising essential elements, including the first author’s name, the year of publication, countries, study design, participants, intervention details, outcome variables, and results (mean± standard deviation [SD] for pretest and posttest of intervention). If the study did not provide the mean ± SD, the median and range and/or interquartile range were extracted to calculate the mean and SD using the following formula: mean ≈ (q1 + m + q3)/3; SD ≈ (q3 − q1)/1.35 [51]. Subsequently, two reviewers (ZS and QY) independently read the full text of the included articles to extract information. A third one (WCL) was consulted for resolution and consensus.

### Data synthesis and analysis

Review Manager 5.3 (Revman) was employed to organize the data and perform the meta-analysis. First, the mean difference (MD) before and after the intervention was calculated. For RCT, if the data of the pretest were not provided, the MD could not be calculated, and the mean of the posttest was used. The MD or mean, SD, and number of participants in the experimental and control groups were entered into Revman to generate the estimated effect size. Subsequently, the effect estimate and its standard error (calculated according to the literature [37]) were entered to generate the final effect size. The standardized mean difference (SMD) is a commonly used effect size metric in meta-analyses that allows for the comparison of studies with different measurement scales or units [25]. The heterogeneity of multiple studies was evaluated using Cochrane’s Q before statistics were merged into the total effect size. I^2^ represents the level of heterogeneity and is based on Higgins’s interpretation of heterogeneity; I^2^ values of approximately 25%, 50%, and 75% indicate low, medium, and high heterogeneity, respectively [25]. The Generic inverse variance method with a random-effects model was applied in the meta-analysis [25].

Sensitivity analysis was performed to identify the extent to which the conclusions of the meta-analysis are influenced by different factors, such as study quality, inclusion criteria, statistical methods, or assumptions made during the analysis (Thabane et al., 2013). In line with the Cochrane Library’s recommendations, given the small number of studies included in our meta-analyses (fewer than 10), assessing publication bias using methods such as funnel plots would be statistically unreliable. The Grades of Recommendation, Assessment, Development, and Evaluation (GRADE) criteria was used to assess the evidence of outcomes [25].

Cohen’s effect size, specifically Cohen’s d = (M2 − M1) ⁄√((SD1^2^ + SD2^2^) ⁄ 2), was calculated to quantify the influence of the intervention in a single study [48]. Cohen’s guidelines were followed, where effect sizes were categorized as small (d-values of 0.2–0.4), medium (d-values of 0.5–0.7), and large (d-values ≥ 0.8) [48]. Moreover, a narrative synthesis was utilized to analyze data that could not be systematically synthesized by this meta-analysis.

## Results

### Selection results

**Fig. 1:**
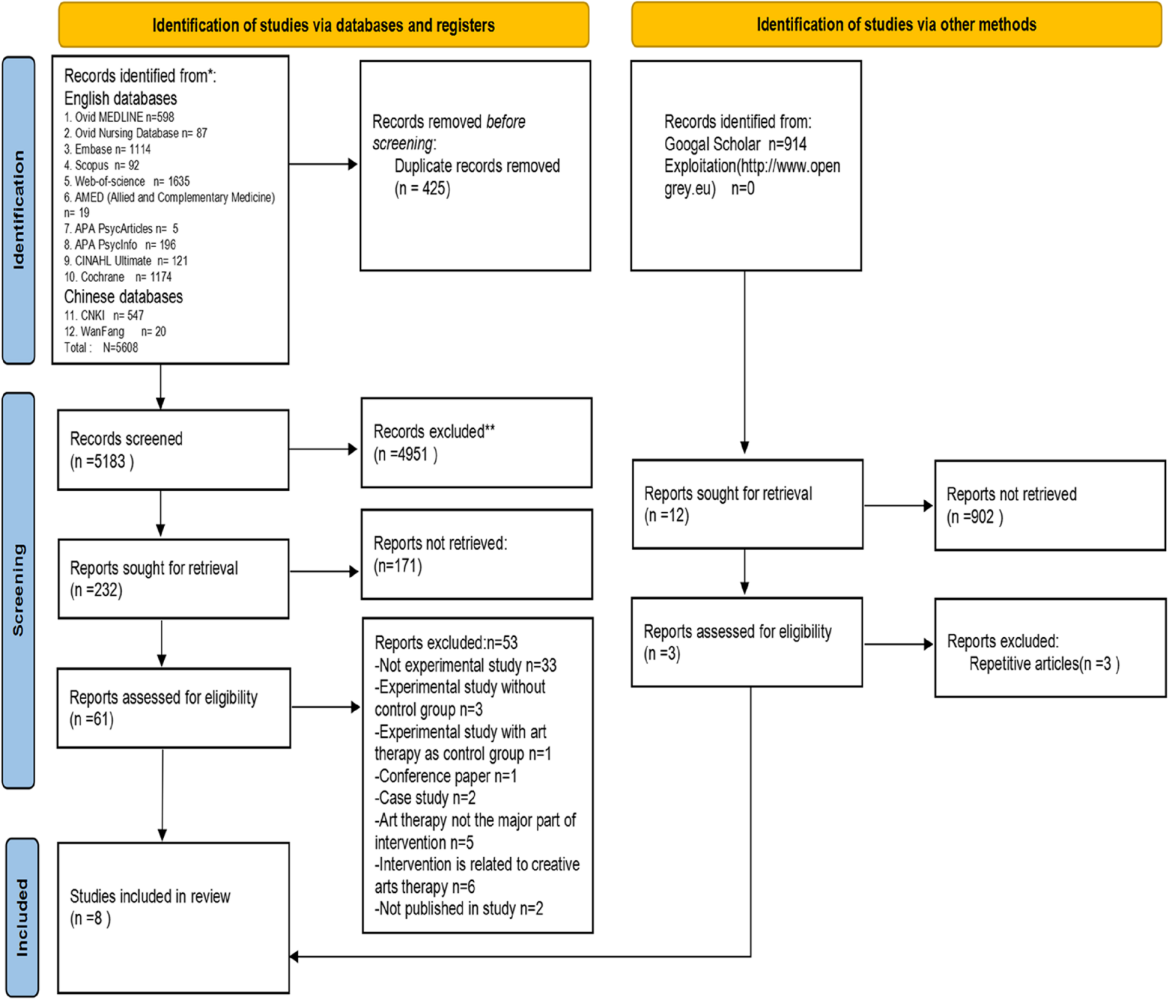
PRISMA flowchart of study selection process and results

The study selection process is illustrated in Figure 1 and adheres to the PRISMA guidelines. A total of 5,608 articles were identified from databases. After duplication, 425 were removed. Following a two-step independent review for title and abstract, 61 articles remained. Subsequently, 53 studies were excluded due to unfulfillment of eligibility. Thereafter, eight articles met the inclusion criteria. A total of 914 studies were retrieved from the grey literature. Following the independent screening process, three eligible studies were identified; however, duplicates were found in eight of the studies. Eventually, eight articles comprising three RCTs and five quasi-experimental studies with a control group were included in this review.

### Quality assessment of included studies

**Fig. 2:**
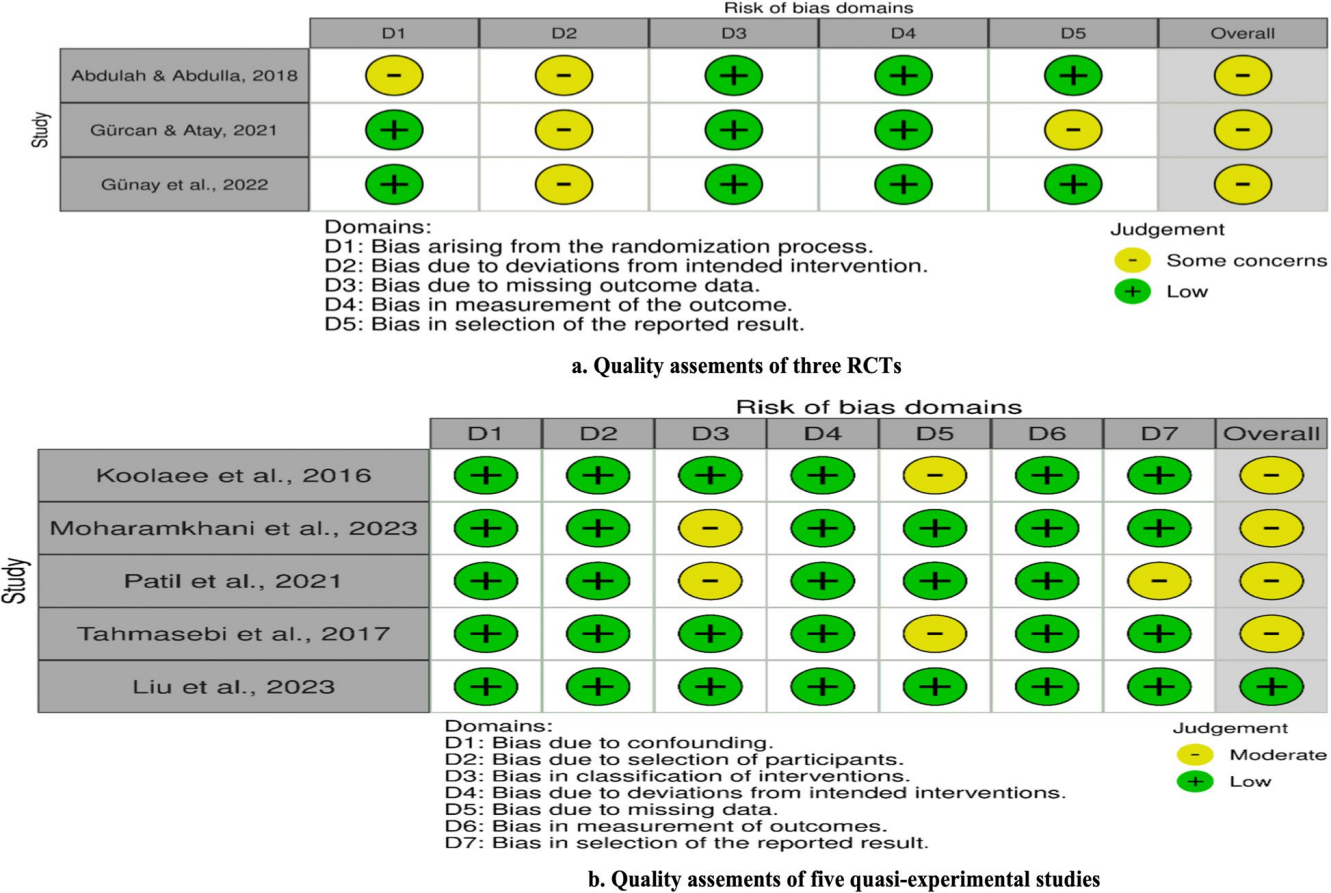
The quality assessment for RCT and quasi-experimental studies

Figure 2 show the assessment of the risk of bias for RCT and quasi-experimental, respectively. All three RCTs [1, 21, 22] had some concerns about the overall quality and bias due to bias arising from the randomization process (D1) with no information on the baseline differences [1], no description of the statistical methods applied in the results (D5) [22], and deviations from the intended intervention (D2) for no words whether deviations arose due to trial context (effects of recruitment and engagement activities on trial participants) [1, 21, 22]. For five experimental studies, one had a low risk of bias [31]. Four were identified as moderate risk without reporting the dropout rate (D5) [29, 46], detailed information on art therapy intervention for the intervention group (D3) [35, 40], and complete outcome results (D7) [40].

### Study characteristics

Characteristics of the eight included studies were extracted and summarized in Table 1, utilizing the Cochrane Handbook as a reference [25]. The eight studies involved a total of 452 participants from five countries: Iraq (*n* = 1), Turkey (*n* = 2), Iran (*n* = 3), India (*n* = 1), and China (*n* =1). The age range of the participants was 7–18 years, and all of them were undergoing cancer treatment while being hospitalized. Inclusion criteria were set for the emotional symptoms indicators in three studies with anxiety and depression reaching a certain score [29, 35, 46]. Only two studies included participants over 14 years old [21, 22]. Five studies had no dropout participants from the control and intervention groups [1, 21, 22, 35, 40]. One study mentioned the dropout rate, including four from the intervention group because of aggravation of the condition and hospital transfer and two in the control group due to hospital transfer and applied and per-protocol analysis method [31]. Two other studies did not report the dropout rate without information for intention-to-trait analysis method [29, 46].

**Table 1:** Characteristics of included study

**Study**	**Country**	**Study Design**	**Sample size**	**Paticipants**	**Control**	**Theory**	**Intervention details**	**Invention implementers Delivery type Settings Materials**	**Intervention dosage: Sessions Time of each Frequence Time of duration**	**Main outcome and results**	**Evaluation tools**	**Measure Time**
[1]	Iraq	RCT	60	hospitalized children undergoing chemotherapy; any type of cancer; Mean age: 9.61 (7-13) years; male: 35, female :25; IG:30, CG:30 Drop out:0	Usual care	None	A mixed model pf painting and art-making: painting based on various desired perspectives of their life and nature; handcraft works based on painting; group discussion with non-violence One of parents are invited to participate in the intervention	· Professional artist · Face to face Group Hospital Watercolour paints, a brush, a pen, and handcrafting materials	20 sessions 2 hours each 5 session every weekFour weeks	**Depressive moods:** ↓ (d=0.80, *P*<0.00)	Kid screen -10 Index Health Questionnaire	Not measured at baseline After intervention Immediately
[21]	Turkey	RCT	62	62 hospitalized children undergoing cancer treatment; any type of cancer with 90.3% leukemia; age:7-18years; male: 20, female :42; IG:32, CG:30 Drop out:0	Usual care	None	Children was taught to how to make the jewelry and make the style they like, helping when necessary	· Rsearchers with experience of jewelry making course Face to face Hospital Individual Beads of various colors and sizes along withjewelry thread, pliers, scissors, and clips	8 sessions 1.5 hours each Two days a week (Monday and Thursday) Four weeks	**Anxiety State of Anxiety** first week: ↓ (d=0.61, *p*=0.00) fourth week ↓ (d=1.47, *p*=0.00) **Trait of Anxiety** forth week ↓ (d=0.84, *p*=0.00))	State-Trait Anxiety Inventory for Children	Before the first time of intervention After the first week intervention immediately Before the last time intervention After the last time intervention immediately
[22]	Turkey	RCT	60	hospitalized children undergoing cancer treatment; any type of cancer with 53.3% leukemias 6; age: 12-17 years; male: 34, female :26; IG:30, CG:30 Drop out:0	Usual care	None	Mandala drawing based on American Art Therapy Association. First Session: Watch mandala videos to learn how to paint and then draw freely while listening to classical or instrumental music. Second session: freely drew and painted a mandala within a not preset circle while listening to music.	· Rsearchers with experience of mandala painting course Face to face Individual Hospital Felt-tip pens (24 different colours), drawing paper, pencils, compasses, ruler, pencil sharpeners and erasers	2 sessions 1-2 hours each An interval of 2 or 3 days 5-6-day	**Anxiety** ↓ (d=0.84, *p*=0.00); **Depression** ↓ (d=0.86, *p*<0.001) **Overall psychological symptoms** ↓ (d=1.24, *p*<0.00) frequency ↓ (d=1.36, *p*<0.00) severity ↓(d=1.40, *p*<0.00) Distress ↓(d=1.32, *p*<0.00)	The Hospital Anxiety and Depression Scale (HADS) The Memorial Symptom Assessment Scale (MSAS)	Before the intervention One day after the intervention
[29]	Iran	Quasi-experimental design with control group	30	hospitalized children; 100% leukemia; Mean age:10.3 (8-12) years; male: 14, female :16; IG:15, CG:15 Drop out:NR	Usual care	None	1. Introduction 2. Collaborative painting 3. Technique of children’s scribble. 4. Photo collage 5. Drawing with free issue 6. Drawing the theme of hospital 7. Drawing family as animal 8. Anger collage 9. Drawing with free issue 10. Evaluate 11. Evaluate	Master in clinical psychology and worked as an art therapist Face to face NR Hospital NR	11 sessions 1 hour each twice per weekfive weeks	**Anger** No significant difference betweem two groups (F=2.36, *p*=0.13) but show efficacy between pretest and posttest (F=118.79, *p*<0.00) **Anxiety** No significant difference (F=3.10, *p* =0.07) but show efficacy between pretest and posttest(F=41.03, *p*<0.001)	The Children's Inventory of Anger (ChIA) The Spence Children's Anxiety Scale	Before the intervention One week after the intervention
[35]	Iran	Quasi-experimental design with control group	80	hospitalized children; any type of cancer; Mean age:11.2 ± 1.79 (9-14) years; male: NR, female :NR; IG:40, CG:40 Drop out:0	Usual care	None	First create relationship with child, then children coloring a mandala design selected from the mandala coloring book Mandala sketches were simple at first and grow more complicated in last sessions	· NR· Face to face· Hospital· Individual Color pencils	6 sessions each 45 min 6 consecutive days	**Anxiety** After the intervention immediately ↓ (d=2.02, *p*<0.05) One month after the intervention ended ↓(d=2.01, *p*<0.05)	Spielberger State-Trait Anxiety Inventory	Before the intervention After the intervention immediately One month after the intervention ended
[40]	India	Quasi-experimental design with control group	30	hospitalized children undergoing cancer treatment any type of cancer; age:7-12years; male: 20, female :10; IG:15, CG:15 Drop out:0	Usual care	None	Drawing, painting and modeling with clay Children can choose one freely at each session	NR Face to face NR Hospital NR	5 sessions, 30 min each, consecutive day five days	**Stress:** NR in the group difference, but repot it decresed in the intervention group from Pretest to Posttest with significant difference (*p*<0.05) **Anxiety:** NR in the group difference, but repot it decresed in the intervention group from pretest to posttest with significant difference (*p* <0.05)	Perceived stress scale-children Hamilton Anxiety Rating Scale (HAM-A)	Before the intervention Two days after the intervention
[46]	Iran	Quasi-experimental design with control group	65	65 hospitalized children undergoing chemotherapy any type of cancer; age:7-12years; male: NR, female :NR; IG:32, CG:33 Drop out: NR	Usual care	None	Open watercolor painting	· NR Face to face Group Hospital· NR	6 sessions 25 min once a week 6 weeks	**Depression** ↓ (d=4.94, *p*<0.00)	Children's Depression Inventory (CDI)	Before the intervention After the intervention immediately
[31]	China	Quasi-experimental design with control group	65	hospitalized children undergoing treatment100% leukemia; Mean age:10years (7-14 years); male: 42, female :32; IG:40, CG:40 Drop out: IG:4, CG:2	Usual care	None	Parent-involved painting and art-making program 1. Encounter with you: Collaborative Painting Relay; House Tree People Painting (Parents Ivolved) 2. Self-Expression: Theme Painting: "Me"; Soft Clay Artwork of My Family (PAC) 3. Emotional Expression: Theme Painting: "My Emotions"; Rainy Figure Painting (Parents Ivolved) 4. Clearing the Mind: Theme Painting: "Little Worries"; "Secret Garden" Coloring (Parents Ivolved) 5. Moving Forward Together: Theme Painting: "My Wishes"; "Me and the Child," "Me and Mom and Dad" (Parents Ivolved) 6. New Journey: Theme Painting: "Bright Future"; Sharing Feelings	· Three nurses finished the course of painting psychoanalyst and one master of nursing students under supervision of psychological consultation Face to face Group Hospital ` A4 paper, erasers, colored pens, clay, etc	6 sessions 45-50 min once a week 6 weeks	**Anxiety** The intervention begins at 3 weeks : ↓(d=0.52, *p*<0.00) After the intervention ended immediately ↓(d=0.90, *p*<0.00); **Depression** no Significant difference at two time point (*p*>0.05)	Screen for Child Anxiety Related Emotional Disorders (SCARED) Depression Self-Rating Scale for Children (DSRSC)	Before the intervention The intervention begins at 3 weeks After the intervention ended immediately

*Note: RCT* Randomised Controled Trial, * NR* not report in the article, *d* Cohen's d

Intervention content details are shown in Table 1. Among eight studies, half applied mixed modalities with painting and handcrafting [1, 29, 31, 40], and the four other studies used a single element.

All interventions in the review were delivered face-to-face in clinical settings. Regarding the format, three studies adopted a group format [1, 31, 46], three employed an individual format [21, 22, 35], and two did not report their format [29, 40]. The dosage could be divided into long-term (4–5 weeks for twice a week, once a week, and five times a week) and short-term interventions (5–6 days with an interval of 2 or 3 days and consecutive days).

Among the studies, one study reported that the participants in the control group were not subjected to any form of intervention [29], whereas participants in the control group from the other included studies received usual care without a detailed description, except in one study that included environmental, medical, and dietary care [31].

### Characteristics of outcomes

The outcomes were primarily categorized into overall psychological symptoms [22], anxiety [21, 22, 29, 31, 35, 40], depression [1, 22, 31, 46], the results of each outcomes can be seen in table 1.

Regarding the evaluation points, nearly all studies evaluated the outcomes at the baseline except one study [1]. Five studies [1, 21, 31, 35, 46] measured the effectiveness of art therapy immediately after the last time of intervention, and other studies evaluated at 1 day [22], 2 days [40], and 1 week [29] after the intervention. Moreover, two studies focused on the effectiveness of the intervention process. Gürcan [22] studied the effects of art therapy after the first week of intervention and before the last time of intervention, and Liu et al. [31] conducted a survey before the intervention began at 3 weeks. The effects of art therapy at the follow-up time were determined by only one study [35] at 1 month after the intervention.

### Effects of art therapy

#### Anxiety

**Fig. 3:**
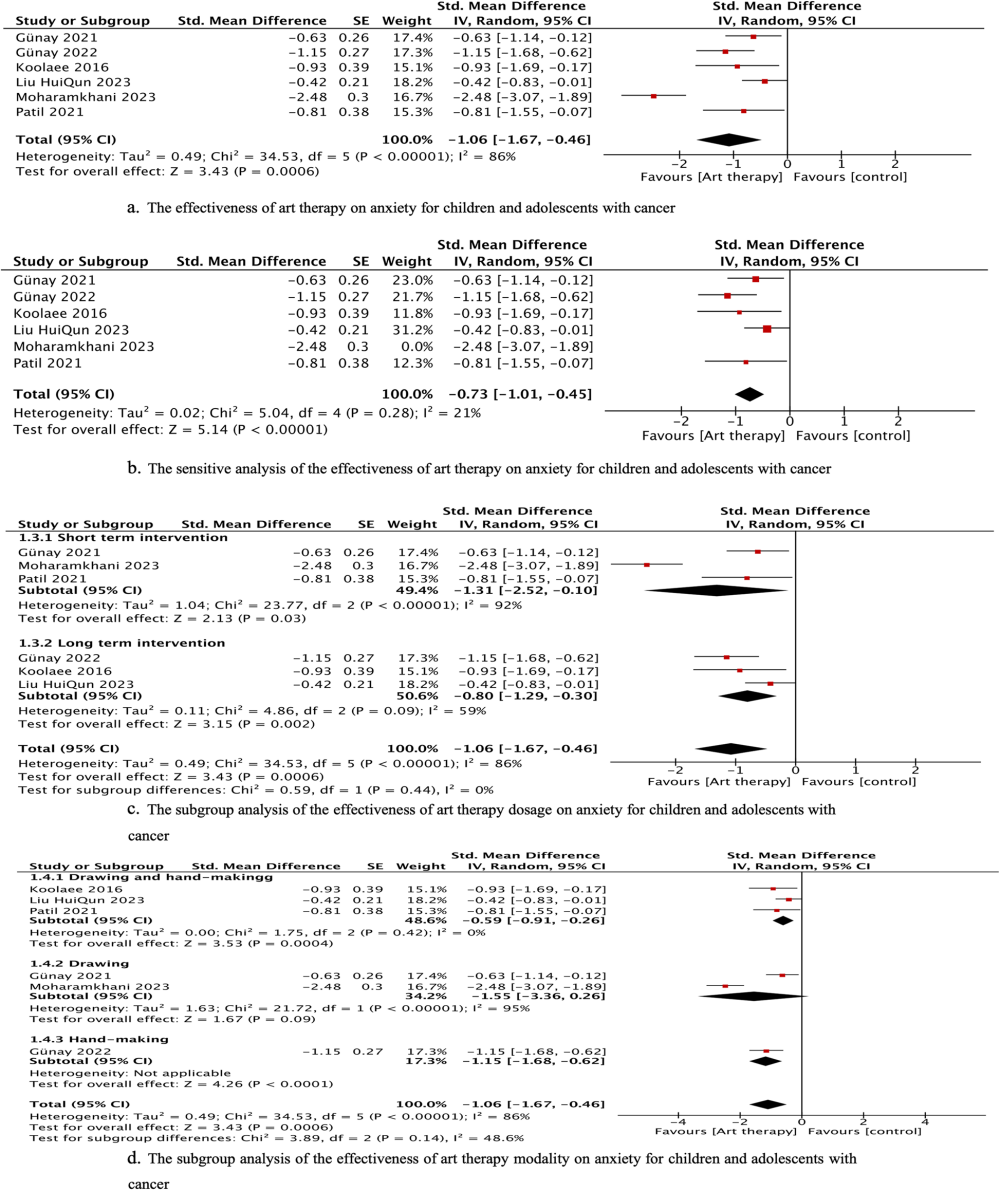
The effectiveness of art therapy on anxiety for children and adolescents with cancer

Our meta-analysis of six studies showed that art therapy significantly decreased anxiety (*p*<0.00, SMD = −1.06, 95% CI = −1.67 to −0.46) with high heterogeneity (I^2^ = 86%, *p* < 0.00) immediately after the intervention (Figure 3a). Among the included studies, the study of Moharamkhani had the highest effect size. According to sensitivity analysis (Figure3b), the results still favored art therapy when this study was removed from the meta-analysis; the effect size changed from −1.06 to −0.73, and the heterogeneity decreased (I^2^ = 21%). Thus, the results were robust, and the high heterogeneity may come from this study. These six studies assessed anxiety outcomes by the State–Trait Anxiety Inventory for Children [21], the Hospital Anxiety and Depression Scale (HADS, [22]), the Spence Children’s Anxiety Scale [29], the Spielberger State–Trait Anxiety Inventory [35], the Hamilton Anxiety Rating Scale [40], and the Screen for Child Anxiety Related Emotional Disorders [31]. Moderate evidence of anxiety was identified.

The subgroup analysis of the intervention dosage for the effectiveness on anxiety among children and adolescents with cancer (Figure 3c) showed that the effect size of the short-term intervention was larger (*p*<0.02, SMD = −1.31, 95% CI = −2.52 to −0.10) than that of the long-term intervention (*p*<0.00, SMD = −0.80, 95% CI = −1.29 to −0.30). Figure 3d demonstrates the results of subgroup analysis of the intervention modality. Among the interventions, drawing had the largest effect size (*p* =0.09, SMD=−1.55, 95% CI = −3.36 to −0.26), but the difference was not significant; this was followed by handcrafting (*p* <0.001, SMD=−1.06, 95% CI = −1.67 to −0.46) and drawing and handcrafting (*p* <0.00, SMD=−0.59, 95% CI = −0,91 to −0.26). These two subgroups also illustrated that the heterogeneity did not come from the intervention dosage and modality.

Moharamkhani (2023) determined the efficacy of art therapy after 1 month, and they found that art therapy positively (Cohen’s d = 2.01, *p*<0.05) reduced trait anxiety for children with cancer (aged 9–14 years old). The intervention of art therapy could also significantly decrease the anxiety of patients with pediatric cancer (Cohen’s d =0.52, *p*<0.001) during the mid-intervention period [31].

#### Depression

**Fig. 4:**
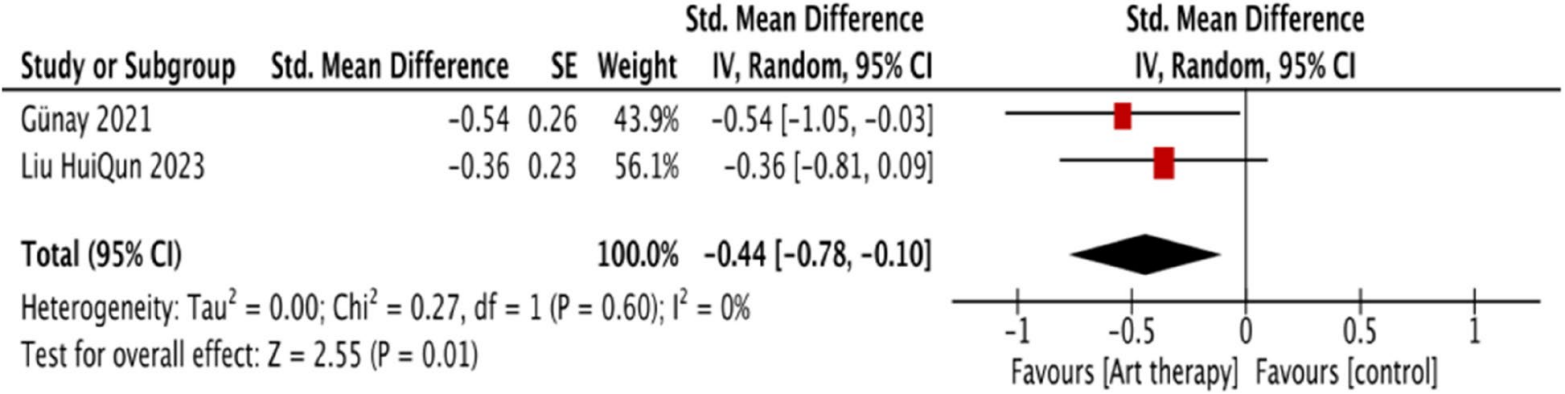
The effectiveness of art therapy on depression for children and adolescents with cancer

The meta- analysis of two studies [22, 31] provided evidence in favor of art therapy, showing a significant difference between the two groups (*p* < 0.01, SMD = −0.44, 95% CI = −0.78 to −0.10) without heterogeneity (I^2^ = 0%, *p* = 0.60; Figure 4). This evidence was identified as moderate one.

Three studies evaluated depression by applying the measurement of HADS [22], the Children's Depression Inventory (CDI; [46]), and the Depression Self-Rating Scale for Children (DSRSC, [31]). Depressive emotions were also evaluated by applying the kid screen-10 Index Health Questionnaire in one RCT with effect size 0.80 (*P*<0.00) [1]. However, a self-contradictory situation was reported in one study by Tahmasebi et al. [46]. The scores of depression in the intervention group after the intervention were higher (mean = 25.7, SD = 3.3) than those in the control group with usual care (mean = 9.9, SD = 3.1). The results section stated that the mean score of depression in the intervention group was significantly lower than that in the control group. The discrepancy in the reporting of depression score outcomes in the intervention group may introduce ambiguity regarding the actual effects. For this reason, this study was not pooled into the meta-analysis.

#### Overall psychological symptoms, stress, and anger

An RCT conducted by Gürcan and Atay [22] evaluated the overall psychological symptoms including six items (difficulty concentrating, difficulty sleeping, feeling sad, feeling worried, feeling irritable, and feeling nervous) by applying the psychological subscale of the Memorial Symptom Assessment Scale . They found that art therapy significantly reduced the overall psychological symptoms (*d* = 1.24, *p* < 0.001), frequency (*d* = 1.36, *p* < 0.001), severity (d = 1.40, *p* < 0.001), and distress (d = 1.32, *p* < 0.00) for the intervention group compared with the control group receiving usual care.

Regarding stress, a quasi-experimental study [40] using the Perceived Stress Scale for Children reported a significant decrease in stress levels in the intervention group (*p* < 0.05) from the pretest to the posttest. However, the intervention and control groups showed no significant difference in stress levels.

In another quasi-experimental study examining anger levels using the questionnaire of Children’s Inventory of Anger (ChIA) [29] in children and adolescents with cancer, they found no significant difference in anger scores between the intervention and control groups (F = 2.36,* p* = 0.13). However, when comparing the pre- and post-test anger scores, a significant difference was observed within the intervention group (*p* = 0.001), but the differences between the intervention and control groups were not significant.

### Quality of the evidence

The quality of evidence for the effects of art therapy on psychological outcomes varies across different measures according to the GRADE approach, as illustrated in Table 2.

**Table 2:** GRADE summary of findings

**Outcome**	**Effect**	**No. of Studies**	**Quality of Evidence**
Anxiety	SMD=-1.06, 95% CI= -1.67, -0.46	6 (2 RCT, 4 quasi-experimental study)	Moderate
Depression	SMD=-0.44, 95% CI= -0.78, -0.10	2 (1 RCT, 4 quasi-experimental study)	Low
Overall psychological symptoms	d=1.24	1 RCT	Low
Anger	No significant difference betweem two groups (F=2.36, *p*=0.13) but show efficacy between pretest and posttest (F=118.79, *p*<0.00)	1Quasi-experimental study	Very low
Stress	NR in the group difference, but repot it decresed in the intervention group from Pretest to Posttest with significant difference (*p*<0.05)	1Quasi-experimental study	Very low

The meta-analysis for anxiety included six studies. While the results favored art therapy, the overall quality of evidence was rated as moderate. This was primarily due to concerns regarding inconsistency (high heterogeneity) and risk of bias in some of the included studies. The meta-analysis for depression was based on only two studies with a potential risk of bias. As a result, the quality of evidence for this outcome was rated as low. Overall psychological symptoms, stress, and anger were each assessed by only a single study (one RCT and two quasi-experimental studies). Therefore, the quality of evidence for overall psychological symptoms is considered low and very low for stress and anger. This is due to the inherent limitations of single-study evidence, including potential biases and imprecision.

## Discussion

This systematic review aimed to investigate the effects of one kind of sensory art therapy, specifically art therapy, on psychological outcomes among patients with pediatric cancer. Additionally, a meta-analysis was conducted to assess the overall effect size of art therapy. While art therapy has gained popularity in treating patients with pediatric cancer, the number of experimental studies determining its effect size remains limited. In this review, only eight studies emphasizing the potential positive impact of art therapy on psychological outcomes were included.

### Effectiveness of art therapy

Our findings suggested that art therapy can positively impact anxiety, depression, overall psychological symptoms, stress, and anger in this population.

### Anxiety

Our meta-analysis of six studies revealed a statistically significant reduction in anxiety among pediatric cancer patients who received art therapy. While considerable heterogeneity was observed across these studies, sensitivity analysis confirmed the robustness of our findings. Specifically, the effect size remained significant (SMD = -0.73) even after excluding the study by Moharamkhani et al. [35], which appeared to be a primary source of the heterogeneity. Subgroup analyses indicated that shorter-term interventions tended to yield larger effect sizes (SMD = -1.31) than their longer-term counterparts (SMD = -0.80). Drawing as a modality demonstrated the largest effect size (SMD = -1.55), but the difference was not statistically significant.

These results align with a growing body of evidence suggesting that art therapy can be an effective intervention for reducing anxiety in both pediatric and adult cancer populations [54]. For instance, a recent meta-analysis found that art therapy significantly reduced both state and trait anxiety in children and adolescents [52]. Moreover, two systematic reviews suggest that drawing as a modality may be particularly beneficial for improving mental health outcomes in cancer patients, although further evidence is needed to confirm this [16, 53]. The heterogeneity observed in our analysis may reflect variations in intervention duration, modalities, and measurement tools, a point also highlighted by other researchers in the field [14]. Although our analyses indicate that both short-term and long-term interventions can effectively decrease anxiety, the optimal art therapy dosage and duration remain unclear and should be confirmed in future research.

The anxiolytic effects of art therapy may be attributable to several factors, including its ability to provide a non-verbal outlet for emotional expression, promote relaxation, enhance self-esteem, and facilitate coping skills [4, 9, 44]. Indeed, one study demonstrated that art therapy can activate the parasympathetic nervous system, leading to a reduction in physiological indicators of anxiety [3]. Considering these findings, incorporating art therapy into the care plan for pediatric cancer patients appears to be a promising strategy for anxiety management, for which our review could only provide moderate evidence.

### Depression

Our meta-analysis of two studies [22, 31] demonstrated a significant effect of art therapy on reducing depression, with no evidence of heterogeneity. However, it is important to note that the study by Tahmasebi et al. [46] reported contradictory results, with higher depression scores in the intervention group compared to the control group. This discrepancy may be due to methodological inconsistencies or reporting bias within that study.

These results are consistent with those observed in adult cancer patients, where art therapy has been shown to significantly reduce depressive symptoms. Similarly, a recent study demonstrated that art therapy can improve depression across various populations, including individuals with moderate-to-severe major depressive disorder, older women, and nursing home residents [50]. Additionally, this research suggested that art therapy can enhance self-awareness and promote emotional regulation, which are crucial for managing depression, further indicating that it is an effective intervention for reducing depressive symptoms, particularly when used in conjunction with other treatments [38]. However, given the limited number of studies included in our meta-analysis, the evidence supporting the use of art therapy for depression alleviation among pediatric cancer patients is currently of low grade. Future research is needed to better understand the mechanisms and outcomes of art therapy for depression in this specific population.

### Overall psychological symptoms, stress, and anger

These outcomes were only reported in single studies, limiting the strength of our conclusions. The quality of evidence for art therapy on improving overall psychological symptoms, stress, and anger ranges from very low to low. One RCT found that art therapy significantly reduced overall psychological symptoms [22], which is corroborated by a meta-analysis reporting improved psychological health in adult cancer patients following art therapy. Two different quasi-experimental studies respectively reported significant reductions in stress and anger within the intervention group [29, 40]. To a certain extent, these results could be supported by two reviews that focus on various types of sensory arts therapy and creative arts therapies in pediatric cancer care, as these concepts encompass more than just art therapy [5, 36].

Similar to the mechanisms by which art therapy alleviates anxiety and depression, its potential benefits for stress and anger may stem from promoting emotional expression and fostering a sense of control [19]. However, further research is needed to explore these potential mechanisms and determine the effectiveness of art therapy in managing these outcomes in pediatric cancer patients.

### Implications for research and practice

The quality of the included studies in this review, as indicated by the risk of bias assessment, is considered acceptable but not high. Some information, such as intervention implementers, format, dosage, and places, needs to be reported elaborately in the articles. This limitation suggests the necessity for high-quality empirical studies with detailed descriptions, particularly RCTs, to advance research in this area. Further research with various kinds of cancer and age groups of participants is necessary to establish robust evidence on the influence of art therapy on the psychological outcomes of pediatric cancer as most of the participants were children with leukemia and aged 7–18 years old in this review. Moreover, the follow-up effects of the art therapy remain unclear and should be investigated in the future.

The potential reason for the effectiveness of art therapy in this review is that arts have healing effects regardless of the implementers [9]. Although art therapist certification from the AATA can be difficult to obtain, the implementers should receive training from arts and psychology as the implementers to guide the art therapy process [20] (5), and such training may influence the effectiveness of art therapy.

It is worthwhile to introduce and apply art therapy in clinical settings. A potential avenue to consider is whether qualified art therapists can train nursing staff in pediatric cancer departments. This would equip them with knowledge and skills in art therapy and help bridge the gap between the demand for art therapy services and the limited availability of qualified art therapists. This collaborative approach may help extend the benefits of art therapy to a significant number of children and adolescents with cancer.

Overall, the results in this review did not establish a clear relationship among the dosage, implementer, and effectiveness of art therapy intervention. Future studies can be designed to explore the relationship among these components, widen the age range of participants, investigate the follow-up effects of art therapy for short-term and long-term interventions.

### Limitations

This review had several limitations that should be acknowledged. First, the limited number of RCTs included in the analysis, particularly the meta-analyses of depression, which only incorporated two studies. As a result, caution must be exercised when interpreting the findings of this review. Additionally, the small sample size and limited culture context of included studies suggest the need to undertake multicenter studies to enhance the robustness of the findings and the nongeneralisability of the results to other countries, particularly Western countries. Then, the studies analysed in this review exhibited several methodological weaknesses, including deviations from the intended intervention [2, 21, 22] and bias in selecting reported results without comparing baseline [2] and detailed intervention information [40]. These limitations diminished the overall quality of the evidence. Lastly, excluding non-English or non-Chinese language studies may have introduced publication bias into this review, as relevant studies published in other languages were not considered.

## Conclusion

Based on the available evidence, art therapy has promising effects on psychological outcomes. This meta-analysis demonstrated satisfactory results of art therapy in decreasing anxiety and depression for children and adolescents with cancer with moderate evidence. Narrative synthesis revealed potential benefits of art therapy in addressing overall psychological outcomes, stress, and anger. However, these results should be interpreted with caution because of the limited number of studies and methodological heterogeneity. Additional experimental studies, particularly rigorous RCTs with large samples, are necessary to confirm the evidence.

## Supplementary Information

Supplementary Material 1.

Supplementary Material 2.

## Data Availability

The datasets used and analyzed in the current study are available from the corresponding authors upon reasonable request.
