# Supplementary material for: Effects of art therapy on psychological outcomes among children and adolescents with cancer: a systematic review and meta-analysis
Source: BMC Complement Med Ther. 2025 Apr 23;25:149. doi: 10.1186/s12906-025-04866-2 (PMC12016200; doi:10.1186/s12906-025-04866-2)
Supplement: Supplementary file 2 — Supplementary Material 2. [file 12906_2025_4866_MOESM2_ESM.pdf]

Supplementary file 1

Search strategies for various databases

English databases

1. Ovid MEDLINE(R) <1946 to November 06, 2023> 598
2. Embase 1910 to Present 1114
3. Scopus 92
4. Web-of-science 1635
5. AMED (Allied and Complementary Medicine) <1985 to October 2023> 19
6. APA PsycArticles Full Text 5
7. APA PsycInfo <1806 to October Week 5 2023> 196
8. CINAHL Ultimate 121
9. Cochrane 1174
10. Ovid Nursing Database <1946 to October Week 4 2023> 87

Chinese databases

11. CNKI 547
12. WanFang 20

Total 5608

Grey Literature

13. Googal Scholar 914

14. Grey Literature Exploitation(http://www.opengrey.eu) 0

1. Ovid MEDLINE(R) <1946 to November 06, 2023>

1 Art Therapy/ or Paint/ or Art/ or Paintings/ or Clay/ or Sculpture/ 23215
2 (art therap* or art mak* or paint* or drawing or mandala or art appreciat* or art product* or sculpture or clay).ab,ti. 66782
3 1 or 2 80065
4 (neoplasm or neoplas* or neoplasias or cancer* or oncolog* or malignan* or carcinoma or tumor* or tumour*).ab,ti. 3482789
5 exp Neoplasms/ 3893992
6 4 or 5 4605922
7 (child* or pediatr* or teen* or adolescen* or youth*).ab,ti. 1815601
8 Child/ or Adolescent/ or Pediatrics/ 3187212
9 7 or 8 3684825
10 3 and 6 and 9 671
11 limit 10 to humans 670
12 limit 11 to english language 598

14. Embase 1910 to Present

1 Art Therapy/ or Paint/ or Art/ or Paintings/ or Clay/ 80458
2 (art therap* or art mak* or paint* or drawing or mandala or art appreciat* or art product* or sculpture or clay).ab,ti. 103540
3 1 or 2 162645
4 (neoplasm or neoplas* or neoplasias or cancer* or oncolog* or malignan* or carcinoma or tumor* or tumour*).ab,ti. 5417210
5 exp Neoplasms/ 5574977
6 4 or 5 6818519
7 (child* or pediatr* or teen* or adolescen* or youth*).ab,ti. 2650027
8 Child/ or Adolescent/ or Pediatrics/ 3121263
9 7 or 8 4005087
10 3 and 6 and 9 1284
11 limit 10 to humans 1192
12 limit 11 to english language 1114

15. Scopus

(TITLE-ABS-KEY (neoplasm OR neoplas* OR neoplasias OR cancer* OR oncolog * OR malignan* OR carcinoma OR tumor* OR tumour*) AND TITLE-ABS-KEY (art AND therap* OR art AND mak* OR paint* OR drawing OR mandala OR art AN D appreciat* OR art AND product* OR sculpture OR clay) AND TITLE-ABS-KEY

(child* OR pediatr* OR teen* OR adolescen* OR youth*)) 92

16. Web-of-science

Web of Science Search Strategy (v0.1)

Database: Web of Science Core Collection

- WOS.SCI: 1961 to 2023
- WOS.AHCI: 1975 to 2023
- WOS.BHCI: 2005 to 2023
- WOS.BSCI: 2005 to 2023
- WOS.ESCI: 2005 to 2023
- WOS.ISTP: 1990 to 2023
- WOS.SSCI: 1956 to 2023
- WOS.ISSHP: 1990 to 2023

Searches:

1: (TI=(art therap* or art mak* or paint* or drawing or mandala or art appreciat* or art product* or sculpture or clay)) OR AB=(art therap* or art mak* or paint* or drawing or mandala or art appreciat* or art product* or sculpture or clay) Results: 1196545

2: (TI=(neoplasm or neoplas* or neoplasias or cancer* or oncolog* or malignan* or carcinoma or tumor* or tumour*)) OR AB=(neoplasm or neoplas* or neoplasias or cancer* or oncolog* or malignan* or carcinoma or tumor* or tumour*) Results: 4482823

3: (TI=(child* or pediatr* or teen* or adolescen* or youth*)) OR AB=(child* or pediatr* or teen* or adolescen* or youth*) Results: 2591066

4: #3 AND #2 AND #1 Results: 1635

17. AMED (Allied and Complementary Medicine) <1985 to October 2023>

1 Art Therapy/ or Art/ 1630
2 (art therap* or art mak* or paint* or drawing or mandala or art appreciat* or art product* or sculpture or clay).ab,ti. 1860
3 1 or 2 2563
4 (neoplasm or neoplas* or neoplasias or cancer* or oncolog* or malignan* or carcinoma or tumor* or tumour*).ab,ti.21496

5 exp Neoplasms/ 18086
6 4 or 5 24994
7 (child* or pediatr* or teen* or adolescen* or youth*).ab,ti. 25652
8 Child/ or Adolescent/ or Pediatrics/ 23092
9 7 or 8 31225
10 3 and 6 and 9 19

18. APA PsycArticles Full Text

1 (art therap* or art mak* or paint* or drawing or mandala or art appreciat* or art product* or sculpture or clay).ab,ti. 2174
2 (neoplasm or neoplas* or neoplasias or cancer* or oncolog* or malignan* or carcinoma or tumor* or tumour*).ab,ti. 1432
3 (child* or pediatr* or teen* or adolescen* or youth*).mp. [mp=title, abstract, full text, caption text] 86643
4 1 and 2 and 3 5

19. APA PsycInfo <1806 to October Week 5 2023>

1 Art Therapy/ or Art/ or Sculpturing/ or Drawing/ 17751
2 (art therap* or art mak* or paint* or drawing or mandala or art appreciat* or art product* or sculpture or clay).ab,ti. 84910
3 1 or 2 93459
4 (neoplasm or neoplas* or neoplasias or cancer* or oncolog* or malignan* or carcinoma or tumor* or tumour*).ab,ti. 95330
5 exp Neoplasms/ 62415
6 4 or 5 99060
7 (child* or pediatr* or teen* or adolescen* or youth*).ab,ti. 1005369
8 Child/ or Adolescent/ or Pediatrics/ 32047
9 7 or 8 1006634
10 3 and 6 and 9 196

20. CINAHL Ultimate

#	Query	Limiters/Expanders	Last Run Via	Results
S13	S10 AND S11 AND S12	Limiters - Full Text Expanders - Apply equivalent subjects Search modes - Boolean/Phrase	Interface - EBSCOhost Research Databases Search Screen - Advanced Search Database - CINAHL Ultimate	121
S12	S3 OR S8 OR S9	Limiters - Full Text Expanders - Apply equivalent subjects Search modes - Boolean/Phrase	Interface - EBSCOhost Research Databases Search Screen - Advanced Search Database - CINAHL Ultimate	432,565
S11	S2 OR S6 OR S7	Limiters - Full Text Expanders - Apply equivalent subjects Search modes - Boolean/Phrase	Interface - EBSCOhost Research Databases Search Screen - Advanced Search Database - CINAHL Ultimate	254,291
S10	S1 OR S4 OR S5	Limiters - Full Text Expanders - Apply equivalent subjects Search modes - Boolean/Phrase	Interface - EBSCOhost Research Databases Search Screen - Advanced Search Database - CINAHL Ultimate	19,824
S9	AB child* or pediatr* or teen* or adolescen* or youth*	Limiters - Full Text Expanders - Apply equivalent subjects Search modes - Boolean/Phrase	Interface - EBSCOhost Research Databases Search Screen - Advanced Search Database - CINAHL Ultimate	243,079
S8	TI child* or pediatr* or teen* or adolescen* or youth*	Limiters - Full Text Expanders - Apply equivalent subjects Search modes - Boolean/Phrase	Interface - EBSCOhost Research Databases Search Screen - Advanced Search Database - CINAHL Ultimate	191,232

S7	AB neoplasm or neoplas* or neoplasias or cancer* or oncolog* or malignan* or carcinoma or tumor* or tumour*	Limiters - Full Text Expanders - Apply equivalent subjects Search modes - Boolean/Phrase	Interface - EBSCOhost Research Databases Search Screen - Advanced Search Database - CINAHL Ultimate	197,963
S6	TI neoplasm or neoplas* or neoplasias or cancer* or oncolog* or malignan*	Limiters - Full Text Expanders - Apply equivalent subjects	Interface - EBSCOhost Research Databases Search Screen - Advanced	169,537
S4	TI art therap* or art mak* or paint* or drawing or mandala or art appreciat* or art product* or sculpture or clay	Limiters - Full Text Expanders - Apply equivalent subjects Search modes - Boolean/Phrase	Interface - EBSCOhost Research Databases Search Screen - Advanced Search Database - CINAHL Ultimate	2,769
S3	MH"Adolescence" or MH" child" or MH" Pediatrics"	Limiters - Full Text Expanders - Apply equivalent subjects Search modes - Boolean/Phrase	Interface - EBSCOhost Research Databases Search Screen - Advanced Search Database - CINAHL Ultimate	297,218
S2	MH"Oncology" or MH" Carcinoma" or MH" Neoplasms"	Limiters - Full Text Expanders - Apply equivalent subjects Search modes -	Interface - EBSCOhost Research Databases Search Screen - Advanced Search Database - CINAHL Ultimate	33,163

	Boolean/Phrase		
S1 MH "Art"or MH "Paint"or MH "Art Therapy" or MH "drawing"	Limiters - Full Text Expanders - Apply equivalent subjects Search modes - Boolean/Phrase	Interface - EBSCOhost Research Databases Search Screen - Advanced Search Database - CINAHL Ultimate	5,562

21. Cochrane

#1 (Art NEXT therap* or art NEXT mak* or paint* or drawing or mandala or art NEXT appreciat* or art NEXT product* or sculpture or clay):ab OR (Art NEXT therap* or art NEXT mak* or paint* or drawing or mandala or art NEXT appreciat* or art NEXT product* or sculpture or clay):ti 5212

#2 [mh “art therapy”] or [mh “art”] or [mh painting] or [mh drawing] or [mh claying] or [mh sculpture] 600

#3 #1 OR #2 5637

#4 [mh child] or [mh pediatrics] or [mh adolescent] 168846

#5 (child* or pediatr* or teen* or adolescen* or youth*):ab 168757

#6 (child* or pediatr* or teen* or adolescen* or youth*):ti 118430

#7 #4 OR #5 OR #6 291841

#8 [mh neoplasm] 113268

#9 (neoplasm or neoplas* or neoplasias or cancer* or oncolog* or malignan* or carcinoma or tumor* or tumour*):ti 162771

#10 (neoplasm or neoplas* or neoplasias or cancer* or oncolog* or malignan* or carcinoma or tumor* or tumour*):ab 210648

#11 #8 OR #9 OR #10 267574

#12 #3 AND #7 AND #11 74

22. Ovid Nursing Database <1946 to October Week 4 2023>

1 Art Therapy/ or Paint/ or Art/ or Paintings/ or Clay/ or Sculpture/ 5108
2 (art therap* or art mak* or paint* or drawing or mandala or art appreciat* or art product* or sculpture or clay).ab,ti. 3869
3 1 or 2 8322
4 (neoplasm or neoplas* or neoplasias or cancer* or oncolog* or malignan* or carcinoma or tumor* or tumour*).ab,ti. 79511
5 exp Neoplasms/ 92075
6 4 or 5 102852
7 (child* or pediatr* or teen* or adolescen* or youth*).ab,ti. 129769
8 Child/ or Adolescent/ or Pediatrics/ 179822
9 7 or 8 198147
10 3 and 6 and 9 110
11 limit 10 to humans 101
12 limit 11 to english language 87

Chinese databases

23. CNKI (知网)

(主題=藝術治療 + 視覺藝術 + 繪畫 + 手工 + 曼陀羅繪畫 + 雕塑 + 陶泥) AND (主題=腫瘤 + 癌症 + 白血病) AND (主題=患兒 + 兒童 + 兒科 + 青少年) 7

(篇關摘=藝術治療 + 視覺藝術 + 繪畫 + 手工 + 曼陀羅繪畫 + 雕塑 + 陶泥) AND (篇關摘=腫瘤 + 癌症) AND (摘要%儿童 + 青少年 + 患兒 + 兒科) 12

(主題=藝術治療 + 視覺藝術 + 繪畫 + 手工 + 曼陀羅繪畫 + 雕塑 + 陶泥) AND (主題=腫瘤 + 癌症 + 白血病) 528

24. WanFang(万方)

(keyWords:艺术治疗 or 绘画 or 视觉艺术 or 手工 or 曼陀罗绘画 or 雕塑 or 陶泥) (KeyWords:肿瘤 or 癌症 or 白血病) (KeyWords:儿童 or 患儿 or 青少年 or 儿科)

Grey Literature

25. Googal Scholar 914

26. Grey Literature Exploitation(http://www.opengrey.eu) 0
